# Supplementary figures and images for: STAG2 mutations in the normal colon induce upregulation of oncogenic pathways in neighbouring wildtype cells
Source: PLoS One. 2025 Oct 15;20(10):pone.0332499.exml. doi: 10.1371/journal.pone.0332499 (PMC12527130; doi:10.1371/journal.pone.0332499)

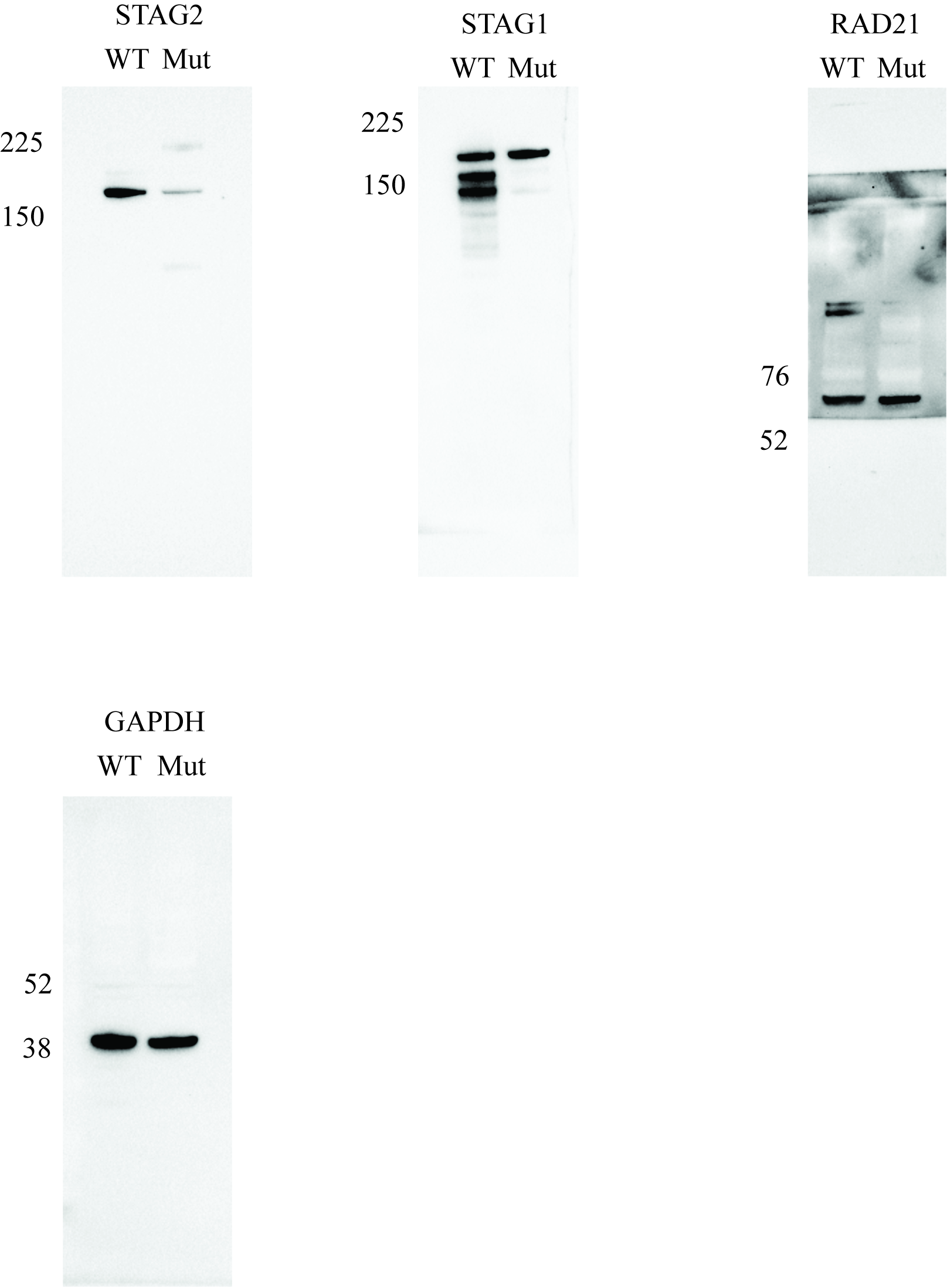

Supplement: S1 Raw Images — (TIF) [file pone.0332499.s003.tif]

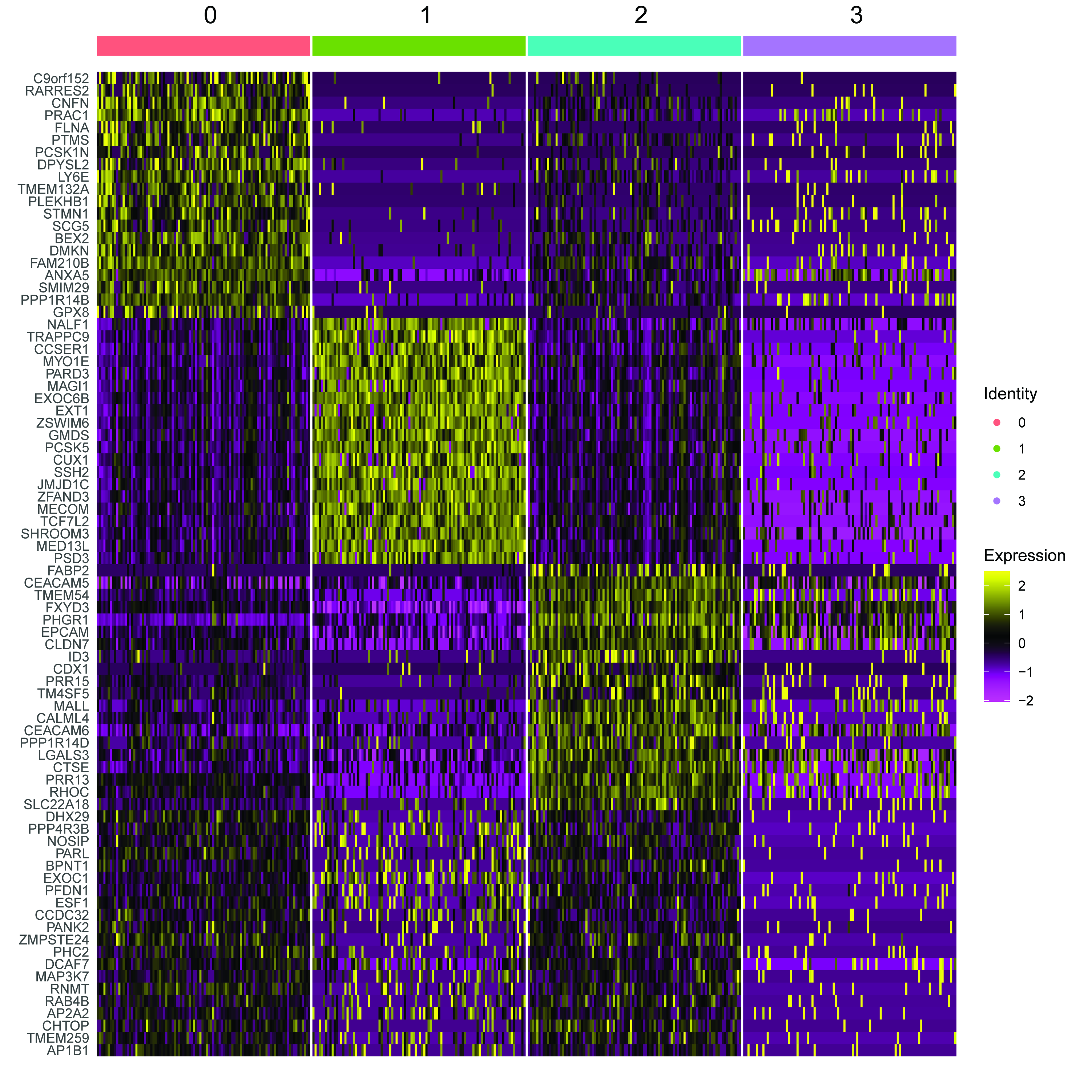

Supplement: S1 Fig — (TIF) [file pone.0332499.s004.tif]
