# Supplementary material for: STAG2 mutations in the normal colon induce upregulation of oncogenic pathways in neighbouring wildtype cells
Source: PLoS One. 2025 Oct 15;20(10):pone.0332499.exml. doi: 10.1371/journal.pone.0332499 (PMC12527130; doi:10.1371/journal.pone.0332499)
Supplement: Attachment — Submitted filename: Reply to PLOS ONE.docx [file pone.0332499.s006.docx]

Dear Editors and Reviewers,

On behalf of the authors, we would like to thank the editors and reviewers for the opportunity to revise our manuscript. The long interim since review was because we took the comments seriously, and performed further experiments to establish the claims in our work. We hope that the editors and reviewers will find these revisions satisfactory.

Please find a point by point reply to the comments made.

Journal requirements

1. We have endeavoured to adhere to PLOS ONE’s styles.
2. Funding statement should read “ Dedrick Kok Hong Chan was supported by an N2CR-CSDU CS Pilot Grant as well as an NUHS Clinician Scientist Program grant (NCSP2.0/2023/NUHS/DCKH).” I have removed the remarks in the Acknowledgement section.
3. Indeed, all sequence reads have already been uploaded in the public domain. I have included the Sequence Read Archive accession number and link in the paper.
4. All western blot uncropped images are found in S1 Fig.
5. We have included tables in the main manuscript file. We also moved Table 2 to Supplementary information.

Reviewer 1

1. We are appreciative that the reviewer felt this was an appropriate approach to investigate our question.
2. We performed additional experiments to address this. In co-cultured organoids, we stained for Ki67 and Cyclin D1, markers of tumorigenicity. We found an upregulation of both in the co-cultured wildtype organoids relative to the *STAG2* mutant organoids. These results are presented.
3. We have included further information about co-culture conditions in the Methods section.
4. We appreciate this comment. To elucidate molecular mechanisms, we performed further immunofluorescent experiments to demonstrate that, in concordance with the sequencing results, upregulation of p53, KRAS and TNFα. Further molecular characterisation will form a future study in which a detailed analysis will be performed. We have included these new results as a new Figure 6.
5. We have included a Figure 8 to demonstrate the findings of this paper in a picture.

Reviewer 2

1. The authors appreciate the highly encouraging comments of this reviewer. Particular, we thank the reviewer for noting that our paper was “very interesting and timely”.

Again, the authors thank the editors and reviewers for this opportunity to revise our paper.

With gratitude,

Dr Dedrick Chan
